# Genetic and Molecular Aspects of Drug-Induced QT Interval Prolongation

**DOI:** 10.3390/ijms22158090

**Published:** 2021-07-28

**Authors:** Daniela Baracaldo-Santamaría, Kevin Llinás-Caballero, Julián Miguel Corso-Ramirez, Carlos Martín Restrepo, Camilo Alberto Dominguez-Dominguez, Dora Janeth Fonseca-Mendoza, Carlos Alberto Calderon-Ospina

**Affiliations:** 1 School of Medicine and Health Sciences, Universidad del Rosario, Bogotá 111221, Colombia; daniela.baracaldo@urosario.edu.co (D.B.-S.); julian.corso@urosario.edu.co (J.M.C.-R.); camilo.dominguez@urosario.edu.co (C.A.D.-D.); 2 GENIUROS Research Group, Center for Research in Genetics and Genomics (CIGGUR), School of Medicine and Health Sciences, Universidad del Rosario, Bogotá 111221, Colombia; kevin.llinas@urosario.edu.co (K.L.-C.); carlos.restrepo@urosario.edu.co (C.M.R.); dora.fonseca@urosario.edu.co (D.J.F.-M.); 3 Institute for Immunological Research, University of Cartagena, Cartagena 130014, Colombia

**Keywords:** pharmacogenetics, pharmacovigilance, drug interactions, arrhythmias, torsades de pointes, long QT syndrome

## Abstract

Long QT syndromes can be either acquired or congenital. Drugs are one of the many etiologies that may induce acquired long QT syndrome. In fact, many drugs frequently used in the clinical setting are a known risk factor for a prolonged QT interval, thus increasing the chances of developing torsade de pointes. The molecular mechanisms involved in the prolongation of the QT interval are common to most medications. However, there is considerable inter-individual variability in drug response, thus making the application of personalized medicine a relevant aspect in long QT syndrome, in order to evaluate the risk of every individual from a pharmacogenetic standpoint.

## 1. Introduction

The association between QT interval prolongation and drug therapy dates back to the 1970–1980s with reports indicating a strange correlation between the use of antibiotics, antipsychotics, and other types of drugs with QT interval prolongation and torsade de pointes (TdP) [1]. Thereafter, in the early 1990s, the United States Food and Drug Administration approved an antihistamine by the name of terfenadine, which later on led to the identification of drug-induced TdP in individuals who were treated with the drug at therapeutic and supratherapeutic doses [2,3].

Consequently, the pro-arrhythmic potential of terfenadine was identified, indicating that at a therapeutic dose it could induce minimal QT interval prolongation, whereas dramatically prolonging the QT interval at a supratherapeutic dose in patients with associated comorbidities (e.g., liver disease) or upon co-administration with drugs that increased terfenadine’s plasma concentration such as ketoconazole (a strong CYP3A4 inhibitor) [4]. Subsequently, fexofenadine—the active metabolite of terfenadine that is responsible for its antihistamine effects—was discovered and terfenadine was withdrawn from the market [1,2]. Since then, the growing interest in understanding and identifying the pro-arrhythmic characteristics of drugs has allowed a broader understanding of the causes and mechanisms of long QT syndrome (LQTS) through molecular genetics.

LQTS is a wide range of cardiac electrophysiological disorders characterized by a prolongation of the QT interval and T wave abnormalities [5]. Prolongation of the QT interval is directly associated with a life-threatening arrhythmia called TdP [6]. The latter consists of a polymorphic ventricular arrhythmia characterized by self-limited bursts that can lead to clinical symptoms such as dizziness, palpitations, syncope, seizures, and, in severe cases, sudden cardiac death [7].

For decades, the duration of ventricular repolarization has been measured using the QT interval. However, this interval is influenced by heart rate. Therefore, it is necessary to correct this measurement by considering the duration of the R-R interval to determine whether a QT interval is normal [8]. The Bazzet formula is used to perform the QT interval correction (QTc) [9]:

(QTc) = Q-T interval (in seconds)/√cardiac cycle (in seconds)

The diagnosis of LQTS can be made by any of the following criteria: QTc interval ≥ 480 ms in 12 repeated leads or a risk score > 3 according to the Scoring System for Clinical Diagnosis of Long QT Syndrome. When unexplained syncope is present, a QTc interval ≥ 460 ms is sufficient to make the diagnosis [10,11]. QT interval is an indirect measure of the ventricular action potential duration [12]. It represents the flow of ion currents through the heart cell membrane via specialized channels formed by protein complexes [13]. Genetic or acquired defects that increase depolarizing Na^+^ and Ca^2+^ currents (I_Na_ and I_Ca_, respectively) or decrease repolarizing K^+^ currents (I_Ks_, I_Kr_, and I_k1_) increase the duration of the action potential and thus causes prolongation of the QT interval [6,14].

The associated refractoriness in cardiomyocytes promotes the formation of early after-depolarizations (EADs) by reactivating L-type Ca^2+^ and Na^+^-Ca^2+^ exchange currents during phases 2 and 3 of the cardiac action potential [14]. It is believed that these focal EADs and the increased electrical heterogeneity in adjacent regions of the myocardium allow the initiation, propagation, and maintenance of TdP.

LQTS can be congenital or acquired. Both entities are a predisposing factor for the development of TdP and increase the risk of sudden death [15]. When mutations occur in the genes coding for the ion channels responsible for the major repolarizing currents, it is considered the congenital form of long QT syndrome (cLQTS) [16]. More than 600 mutations have been identified in the 17 associated genes [16,17]. LQT1 and LQT2 are associated with mutations in the potassium voltage-gated subfamily Q member 1 (*KCNQ1*) and potassium voltage-gated channel subfamily H member 2 (*KCNH2*) genes, respectively; whereas LQT3 is linked with mutations in the sodium voltage-gated channel alpha subunit 5 (*SCN5A*) gene that codes for the alpha subunit of the sodium channel Nav1.5 [18].

On the other hand, acquired long QT syndrome (aLQTS) has been associated with multiple risk factors. Electrolyte abnormalities (e.g., hypokalemia, hypomagnesemia), bradycardia, heart disease, use of certain drugs, drug-drug interactions, subarachnoid hemorrhage, malnutrition, immune deficiency virus (HIV), and genetic predisposition are considered factors that facilitate the development of QT interval prolongation and TdP [10,15,19]. The most common cause of aLQTS is attributed to the use of certain drugs widely used within the clinical setting such as antihistamines, antibiotics, antidepressants, and prokinetics [19].

Determining the incidence or prevalence of drug-induced long QT syndrome (diLQTS) in its entirety is difficult because it is often transitory and the diagnosis requires the recording of an electrocardiogram (ECG) during the episode to determine the prolongation of the QT interval [20]. However, some European pharmacovigilance studies place an annual diLQTS or TdP report at approximately 0.8 to 1.2 per million cases per year [21].

The risk of diLQTS is largely determined by the drug’s interaction with a variety of cardiac ion channels. Thus, variants in the genes encoding cardiac ion channel subunits can modify the drug-channel interaction, increasing its binding affinity or its gating kinetics. Consequently, drugs have the potential to modify the net repolarizing currents in the ventricular action potential, causing prolongation of the QT interval as the final manifestation [7,12]. Likewise, not all patients exposed to the same drug will manifest prolongation of the QT interval, suggesting that diLQTS also has pharmacogenetic determinants that must be taken into account. Genetic variants affecting the drug’s pharmacokinetics and pharmacodynamics will alter the patient’s risk to develop diLQTS [12,22]. Given the growing interest in knowing the risk of drug-induced TdP, a federal grant was issued by the United States government. The aim was to evaluate the available evidence on drugs with a high relative risk of causing TdP and to provide the scientific and medical community with the findings free of charge [20]. This initiative, under the name CredibleMeds^®^, contains a list of more than 100 drugs associated with TdP [23,24].

Similarly, regulatory agencies have adopted International Conference for Harmonization (ICH) guidelines S7B and E14 to establish the pro-arrhythmic characteristics of drugs before they are marketed, these guidelines focus on the reduction of repolarizing cardiac ionic currents and the prolongation of the QT interval induced by drugs. These efforts demonstrate the importance that providing pro-arrhythmic cardiac safety considerations in the development and use of therapeutic measures has in the field of pharmacology [25].

The aim of this article is to highlight the molecular and genetic aspects of aLQTS, given that diLQTS represents an unacceptable risk of sudden death in patients receiving treatment for non-life-threatening conditions [26]. There is also the issue of new, potentially useful drugs being withdrawn from the market due to a small number of TdP cases [26]. Besides, there is no tool available to quantify the exact risk of a drug’s potential to induce aLQTS or TdP, and the development of a better-integrated approach to assess the torsadogenic risk of new drugs is required [27]. Furthermore, there is a compelling need to identify genetic markers of diLQTS and drug-induced TdP susceptibility, in order to guide genotyping and genetic data analysis, as well as to deliver a personalized medicine approach that allows for reductions in the incidence of these adverse drug reactions.

## 2. Congenital Long QT Syndromes

Even though the focus of this review is diLQTS, we intend to make a brief description of cLQTS, as well as the genes involved in these entities. cLQTS are estimated to occur in 1 out of 2500 people; however, prevalence estimations may be hindered by concealed LQTS (i.e., patients that carry the mutation but have normal QTc) [28]. Proposed diagnostic criteria rely mostly on electrocardiographic, clinical, and genetic factors [29]. Moreover, the Schwartz score is also used for cLQTS diagnosis: when a patient has a score of 3.5 points or greater, the probability of having this condition is considered to be high [30]. Generally speaking, management of cLQTS is based on non-pharmacological interventions, such as avoiding QT-prolonging drugs, as well as pharmacological therapies such as beta- or sodium channel blockers and procedures such as left cardiac sympathetic denervation or implantable cardioverter-defibrillator in particular cases [29]. The treatment must be tailored for each patient considering the presence and timing of symptoms, cLQTS subtype, past medical history, occupations/hobbies (competitive athletes), QT interval duration, contraindications for certain drugs or procedures, effectiveness and tolerance of previous therapeutic attempts, and patient’s preferences.

cLQTS are caused by genetic mutations that disrupt normal cardiac function; more specifically, its electrical activity. These mutations mostly occur within genes encoding ion channels—proteins responsible for the selective passive transport of ions, primarily Na^+^ and K^+^—from one side of the myocyte membrane to the other. Alterations in these proteins cause abnormal cardiac ionic currents leading to longer action potentials and further pathophysiological events that may evolve to a life-threatening TdP. These derangements thus carry potentially fatal arrhythmias that can manifest clinically as syncope and/or seizures, as well as delayed ventricular repolarization as evidenced by electrocardiographic QT interval prolongation [18]. Hence, cLQTS is considered a cardiac ion channelopathy.

Genetic heterogeneity is a cardinal feature of cLQTS. These syndromes are categorized into 15 subtypes (LQT1-15), being LQT1-3 the most common ones, but each subtype is caused by polymorphisms in different loci [18]. cLQTS subtypes are mostly inherited in an autosomal dominant fashion, and these are referred to as Romano–Ward syndrome. On the other hand, a severe, autosomal recessive form of cLQTS associated with deafness is known as Jervell and Lange–Nielsen syndrome [28]. Even though around 17 genes have been linked to cLQTS, over 600 mutations have been identified in these genes, reflecting an important mutational heterogeneity [28,31]. However, a study evaluating the available evidence for the 17 reported cLQTS-causative genes yielded interesting results [31]. Following the selection of genes, three independent curation teams assessed and scored the level of evidence for each one of these genes concerning cLQTS. According to their analyses, only three genes (*KCNQ1*, *KCNH2*, and *SCN5A*) have definitive evidence for causing typical cLQTS [31]. These genes are the widely known genetic basis for the three most frequent cLQTS subtypes (LQTS 1-3) and, not surprisingly, were also included in all of the 36 genetic panels for cLQTS included in this study [31]. On the other hand, four genes have definitive/strong evidence for atypical cLQTS [31]. The remaining 10 genes have either moderate (calcium voltage-gated channel subunit alpha1 C, *CACNA1C*) or limited/disputed evidence as for their role in causing cLQTS [31]. These data should be considered when performing genetic testing for this entity. Hereafter, we will focus on the three most frequent subtypes (for information on all the subtypes, see Table 1). Risk stratification and genotype-phenotype correlations are important issues to consider while approaching these patients; nevertheless, these topics are beyond the scope of this review.

Both LQT1 and LQT2 are associated with genes encoding potassium voltage-gated channels involved in the repolarization of heart myocytes during their action potential, while LQT3 is caused by mutations in a sodium voltage-gated channel [18]. The first and most common type of cLQTS, LQT1, is related to a gene located in chromosome 11p, named *KCNQ1*, which encodes for Kv7.1. In LQT1, loss-of-function mutations of this gene decrease the slow delayed rectifier current. In order of frequency, LQT1 is followed by LQT2, which originates from loss-of-function mutations in the gene *KCNH2*, which produces a protein called human Ether-á-go-go-related gene (hERG, a subunit of Kv11.1, a voltage-gated potassium channel) that participates in the rapid delayed rectifier current of the cardiac action potential. Finally, gain-of-function mutations in *SCN5A*, a gene located in the short arm of chromosome 3 coding for the alpha subunit Nav1.5 protein, are responsible for the LQT3 subtype. Because of these mutations, the voltage-dependent sodium current is potentiated in this subtype by incomplete Nav1.5 inactivation [28]. Phenotypic overlap between LQT3 and Brugada syndrome has been described in some patients, as alterations in the Nav1.5 channel are associated with both conditions as well as other cardiac diseases [32]. Nowadays, cutting-edge approaches (e.g., next-generation sequencing and novel computational methods), as well as animal and expression models, are being used to identify and validate new genetic variants causing cLQTS [32].

## 3. Acquired Long QT Syndrome

aLQTS can be defined as a pathological prolongation of the QTc interval, generally greater than 480 ms [26] which is secondary to an environmental trigger (e.g., electrolyte abnormalities, drug therapy) and reverts back to normal upon its withdrawal [34]. The most common cause of aLQTS syndrome is drug therapy, which generates a problem from a pharmacovigilance viewpoint because many drugs (terfenadine, astemizole, grepafloxacin, cisapride) [35] were withdrawn from the market due to QT prolongation [36]. Several drug classes have been associated with this phenomenon including antiarrhythmic drugs, anti-infective medications, antineoplastic, antihistamines, psychotropic drugs, among others [35] whose mechanisms will be discussed later. 

The attention given to aLQTS is due to the risk of developing fatal arrhythmias, especially TdP ventricular tachycardia which increases the risk of sudden death. Furthermore, at the beginning of the severe acute respiratory syndrome coronavirus 2 (SARS-CoV-2) pandemic in early 2020, several drugs known to cause QT prolongation were used concomitantly in the treatment of COVID-19 (e.g., chloroquine, hydroxychloroquine, azithromycin, lopinavir/ritonavir) [37], which raises concern about our knowledge of drug-drug interactions and the correct and safe use of these drugs [38,39]. Nevertheless, aLQTS has not only been associated with drug therapy. Other known risk factors for developing this syndrome include metabolic disorders (e.g., hypokalemia, hypocalcemia, starvation), genetic susceptibility [26], and others such as female sex, heart disease (e.g., myocardial infarction, congestive heart failure), polypharmacy, advanced age, and bradycardia [10], which will be reviewed later on.

### 3.1. Pathophysiology of Drug-Induced Long QT Syndrome

As mentioned previously, a variety of drugs from different pharmacological groups have been associated with prolongation of the QT interval and increased risk of TdP [35]. Generally speaking, the QT interval represents cardiac repolarization, which is regulated by a balance between inward currents (through calcium and sodium channels) and outward currents (through rapid and slow delayed rectifier potassium channels) [1]. QT prolongation results from an increase in inward (depolarizing) currents or a decrease in outward (repolarizing) currents leading to action potential prolongation that manifests in the ECG as QT interval prolongation [1]. The proposed mechanism whereby drugs produce prolongation of the QT interval is through inhibition of the outward rapid delayed rectifier potassium current (I_Kr_), which prolongs individual cardiac ventricular action potentials. This renders individual cardiac cells susceptible to develop EADs, which are the substrate for developing TdP, via phase 2 reentry [1,35]. To better understand the molecular aspects involved in diLQTS, we first need to review the basic properties of cardiac repolarization.

#### 3.1.1. Basic Properties

Myocardial action potentials are the result of activation and inactivation of inward (Na^+^ and Ca^2+^) and outward (K^+^) currents. There are multiple types of voltage-gated K currents mediated by different potassium channels: two transient outward currents, I_to,f_ (transient outward potassium current- fast) and I_to,s_ (transient outward potassium current- slow); and two distinct components of the delayed rectifier potassium current, the rapid component (I_Kr_) and the slow component (I_Ks_) [40]. Cardiac repolarization is principally determined by the I_Ks_ and I_Kr_ and by a final inward rectifier potassium current (I_K1_), which is activated during late repolarization and plays a role in the maintenance of the negative resting potential (phase 4). Cardiac repolarization is a complex process that depends on the correct function of multiple channels. Due to the multiple ionic currents involved in this process, a ’repolarization reserve‘ hypothesis has been linked to different patient’s susceptibility to developing TdP [40] upon exposure to an environmental trigger. The multichannel implications in cardiac repolarization imply a ‘reserve‘ or ’reinforcement‘ in which damage to one component of the repolarization machinery (e.g., loss-of-function mutation of *KCNQ1*) would result in a subclinical presentation (normal QT interval) because the other components would maintain an adequate repolarization mechanism [1]. Nevertheless, if the patient is exposed to a trigger causing QT prolongation (e.g., drugs, hypokalemia) it would unmask such subclinical lesions, leading to significant QT prolongation, EADs, and TdP. Thus, the repolarization reserve is principally modulated by individual genetic characteristics [41].

Another known factor intrinsic to the ventricular myocardium that can predispose to the development of ventricular arrhythmia is its electrical heterogeneity. Functionally, cardiomyocytes can be divided into three types—endocardium, M, and epicardium cells—that confer electrical heterogeneity to ventricle repolarization [42]. This electrical heterogeneity has been associated with various arrhythmias, including TdP. The duration of action potentials among these cells can vary; for example, the action potentials of epicardial and M cells exhibit a notable I_to_—mediated phase 1 that is not seen in endocardial cells. Additionally, M cell action potentials tend to be longer due to smaller I_Ks_ and a larger I_Na_ (sodium current) and sodium-calcium exchange current compared to the other cardiac cells [42]. In the ECG, these voltage differences are responsible for the T wave, in which the peak of the T wave represents repolarization of the epicardial cells and the end of the T wave coincides with the repolarization of the M cells [42]. Therefore, the QT interval is determined by the duration of the M cell action potential. The importance of this phenomenon lies in the fact that M cells are more susceptible to a variety of medications that block I_Kr_ or I_Ks_, or increase I_Ca_ or I_Na_; increasing the probability of developing a fatal arrhythmia [34,42].

#### 3.1.2. Molecular Aspects of diLQTS

Many drugs with no structural similarities (antihistamines, antipsychotics, antibiotics) share a common mechanism by which they cause diLQTS and TdP. This shared mechanism has been identified as blockade of the I_Kr_ on the cardiac action potential carried out by a voltage-gated potassium channel called Kv11.1. The alpha subunits of this channel are encoded by the *KCNH2* gene [34,43]. There are various voltage-gated potassium channels, usually subdivided into families. The Kv11.x family has three members—Kv11.1, Kv11.2, and Kv11.3—of which Kv11.1 has been implicated in diLQTS, as it is responsible for the I_Kr_ [44]. This blockade results in QT prolongation and increased cardiac repolarization heterogeneity due to M cell susceptibility to drug blockade, as discussed previously. As a result of QT prolongation and transmural heterogeneity in repolarization, EADs may occur, and they are the predisposing factor triggering ventricular arrhythmias (discussed under ’The Role of EADs in diLQTS‘). This pharmacological disruption of cardiac repolarization is widely simulated to predict drug proarrhythmic risk in preclinical stages of drug development, mainly using the ICH guidelines [43]. Nevertheless, several factors affecting the drug/Kv11.1 interaction cannot always be accounted for in simulations [43]. Given the clinically relevant aspects of the Kv11.1 channel, it has been extensively studied.

**Figure 1:**
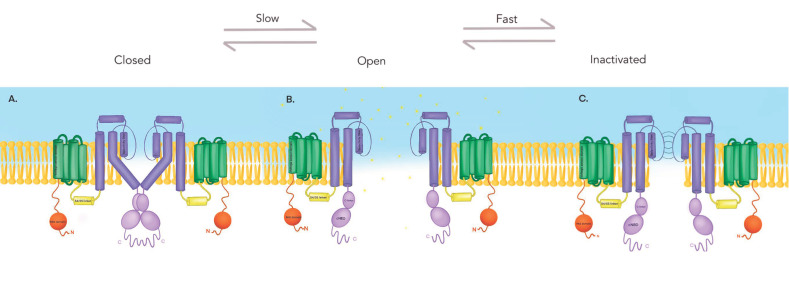
Gating kinetics of the Kv11.1 channel. The Kv11.1 channel can exist in 3 states, open, inactivated, and closed. An important characteristic defining Kv11.1 is its rapid inactivation and recovery from inactivation, as opposed to the slow process of deactivation (closure). Figure adapted from [44]. (**A**) Kv11.1 in the closed state, the four S6 domains crisscross near the cytoplasmic domain to narrow the opening and avoid ion flow. Contains a voltage sensor domain (green) encompassed in the first four transmembrane helices (S1–S4), a pore (dark purple) that serves as the conducting ionic potassium pathway. Each alpha subunit of this tetrameric channel (only two subunits are shown) contains an amino terminus (orange) and a carboxyl terminus (light purple), C-linker and cNBD (cyclic nucleotide-binding homology domain). Figure adapted from: [44] (**B**) Kv11.1 in the open state, the four S6 domains spread outwards to allow the passage of potassium ions. Figure adapted from: [44]. (**C**) Kv11.1 in the inactive state.

The Kv11.1 channel is a homotetramer, composed of four α-subunits all encoded by the same gene (*KCNH2*) [45]. The *KCNH2* gene on chromosome 7, has also been associated with cLQTS, especially LQT 2 [44]. *KCNH2* codes for hERG, a protein of 1,159 amino acids, which is a member of the family of voltage-gated potassium channels. Each hERG protein (or a subunit) has six transmembrane domains (S1–S6) with their respective intracellular amino and carboxyl termini [46] (Figure 1A). The S1–S4 domains form the voltage sensor domain (VSD), which responds to transmembrane voltage changes; whereas the S5 and S6 helices form the pore and the selectivity filter [46]. The completely assembled channel has all four pore domains facing the center, surrounding the ion conduction pathway [44,45,46]. Furthermore, the amino terminus contains a Per-Arnt-Sim (PAS) domain [46,47] a characteristic feature of the voltage-gated potassium channels thought to play a role in its deactivation [44,45,46,48], and the carboxyl terminus contains a cyclic nucleotide-binding domain (cNBD). Even though they share similarities with some other voltage-gated potassium channels, Kv11.1 exhibits some unique inactivation kinetics [44,46].

Kv11.1 channel can adopt a conformation of closed, open, or inactivated state. The gating kinetics in the different states of the potassium channel have been extensively studied, indicating that Kv11.1 elicits unusual gating kinetics compared to other voltage-gated potassium channels, in that inactivation occurs at a faster rate than activation and deactivation (closure), and is a voltage-dependent process [45,48,49]. This means that the transition between open and closed states is very slow, and the transition between open and inactivated states is very fast. These gating characteristics explain the physiological role of I_Kr_ in cardiac repolarization, in that it determines the duration of the plateau phase and, therefore, of the action potential [43,45,49]. As a consequence of slow activation and rapid inactivation, there is little current flow during phases 1 and 2 of the action potential, which contributes to the maintenance of the plateau phase, thereby allowing enough time for Ca^2+^ to enter the cell. As phase 3 ensues, Kv11.1 channels recover from inactivation, increasing the outward current, with the peak outflow at −40 mV [45]. As repolarization ends, channels close slowly, and so they remain open for some time while membrane potential has already returned to the basal state [44]. This phenomenon also contributes to the refractoriness of cardiac cells following repolarization and serves as a mechanism that counteracts possible premature beats [43,44,50]. Hence, if I_Kr_ is blocked, patients are more prone to arrhythmias that are initiated by premature beats.

#### 3.1.3. Kv11.1 Gating Kinetics 

Activation occurs as a result of changes in the transmembrane voltage, which results in an external displacement of the VSD encompassed in the first four transmembrane helices (S1–S4) [46]. As a consequence of this movement, the activation gate (formed by regions of S6 helices) opens at the entrance of the channel pore. The VSD movement is thought to be physically coupled to the movement of the S4/S5 linker (yellow cylinder in Figure 1) and therefore transfers this kinetic energy to the opening and closing of the activation gate. Indeed, recent studies on gating mechanisms of Kv11.1 have supported that the S4/S5 linker serves as a regulator of the gating apparatus in Kv11.1, linking the voltage sensor activation with pore opening through the S4/S5 linker [49].

In its closed state, the activation gate adopts a conformation in which the S6 transmembrane helices are crossing over (Figure 1A) so that it does not allow K^+^ ions to flow. During the open state (Figure 1B), the opposite happens: the helices splay outwards, bending at the hinge to allow the passage of K^+^ ions. The majority of voltage-gated potassium channels have a proline-valine-proline sequence at this hinge; however, the Kv11.1 has a glycine at the position of the second proline (Gly657), relevant to the closing kinetics. Changes in these amino acids have been shown to destabilize the closed formation, therefore favor the open state [50]. As an example, a mutation causing the change of a Gly657 residue for a proline residue causes permanently open channels [45]. Furthermore, the slow deactivation (closure) kinetics has been linked to the NH2 terminal and the PAS domain, in that the first 25 amino acids of the NH2 tail contain a helical element which disruption dramatically accelerates deactivation. It has been hypothesized that the PAS domain establishes a stable relationship with the pore of the channel, bringing the amino-terminal close to the conduction pathway, allowing regulation of the activation gate [44]. Additionally, S4/S5 linker mutations affect the deactivation process, whereby the replacement of cysteine by glycine in position 546 accelerated deactivation, similarly to a deletion of the amino terminus. 

As mentioned, an important characteristic of the Kv11.1 channel relies on marked rapid channel inactivation and recovery from inactivation, and its characteristic voltage dependence. In general, there are two mechanisms for channel inactivation: N-type and C-type inactivation [44]. It is thought that the Kv11.1 channel exhibits C-type inactivation, in which ion flow is blocked by conformational changes in the selectivity filter (where the channel discriminates K^+^ from Na^+^), and it is sensitive to the extracellular potassium concentration. There are two important residues found to be critical for the inactivation process, Ser620 and Ser631 in the pore domain. Mutations in both these residues (*KCNH2* p.Ser631Ala and p.Ser620Thr) show inactivation abolishment and a shift of the V_0.5_ for inactivation by 100 mV. The fast nature of this process has been recently attributed to an asymmetrical constricted-like conformation of the selectivity filter using molecular dynamics simulations [51]. This study linked the side-chain orientation of Phe627 directly to the constriction of the selectivity filter, thus providing an atomistic explanation for the mutations affecting inactivation. Therefore, most of the mutations that influence inactivation facilitate or block the side-chain rotation of Phe627 [51].

#### 3.1.4. Molecular Aspects of Drug/Kv11.1 Interaction

Apart from the unique gating characteristics of the Kv11.1 channel, it also appears to be the only voltage-gated potassium channel that has a profound tropism for drugs. It appears that Kv11.1 has special amino acid residues in the S6 helix and in the pore domain that confer this characteristic. Many mechanisms have been proposed, including a drug-binding pocket, drug trapping mechanisms and a state-dependent binding [45].

The major molecular determinants in the channel pore domain are the aromatic residues (Phe656) and (Tyr652) located on the S6 helix as well as other residues at the base of the selectivity filter (Thr623, Ser624, Val625) [43,44,52] The aromatic residues are particularly important for high-affinity drug block, observed initially in cisapride and dofetilide interaction with the channel due to pi-stacking interactions [53] between the aromatic groups on the blocker compounds and the aromatic residues at the central pore of the channel [54,55].

On the other hand, some inactivation-deficient Kv11.1 mutant variants exhibit reduced sensitivity to drug blockage [56], however, the conclusion that drugs bind more strongly to the inactivated state of the channel is not supported given contradictory study results, for example, increased drug binding of methane sulfonamides to the mutant inactivation-deficient hERG channel, or the fact that inactivation deficient channels do not reduce drug blockage equally [56]. Consequently, this led to the discussion about state-dependent drug blockade, which could manifest different binding kinetics depending on the channel state. The majority of drugs that bind to Kv11.1 are thought to require channel opening in order to gain access to the inner cavity of the channel pore where the important residues reside. However, some compounds do exhibit a greater affinity depending on the state of the channel [43]. An example of this state-dependent blockade was shown by the BeKm-1 scorpion toxin, which exhibits high selectivity and affinity for hERG channels in the closed state. BeKm-1 blocks the channel through a pore obstruction mechanism, with subsequent proarrhythmic effects in a rabbit’s heart [57]. Nevertheless, the majority of drugs that exhibit high-affinity blockade show preferential binding to the inactivated state. The conformational changes required for inactivation may alter the organization of the aromatic and polar residues in the inner cavity that allow some drugs to bind preferentially to the inactivated state [43]. Despite this, there are also non-state-dependent drugs, whose binding is not affected by channel state (quinidine, erythromycin, clozapine) [43].

Moreover, as a result of Ala-scanning-mutagenesis studies that identified the amino acid residues critical for Kv11.1/drug (MK99, a class III anti-arrhythmic) interaction, as well as of pharmacophore models, four deep hydrophobic blocker binding pockets below the selectivity filter have been identified [46]. These hydrophobic ‘pockets’ are oriented away from the central pore cavity and are lined by certain residues (Phe557 on the S5 helix), whose mutations (*KCNH2* p.Phe557Leu) have shown to attenuate the effect of some hERG blockers [46,58,59]. Furthermore, this Kv11.1/drug interaction is also the result of residues found on the S6 helix (Phe656, Tyr652) and residues on the selectivity filter (T623, S625, and V625) [60]. Thus, these regions are one of the possible reasons why Kv11.1 channel exhibits a promiscuous nature to a variety of small molecules. It seems that molecules with high affinity to block adopt a Y-shaped binding mode conformation, positioning the basic amine below the selectivity filter in the ion permeation pathway [60], in contrast to the weak blockage affinity/non-blocker compounds, which cannot adopt this conformation. On the other hand, Kv11.1 activators have also been identified, which could be a beneficial treatment target for LQTS. Kv11.1 activators could have a binding pocket that overlaps with that of blockers, which opens the channel and increase K outflow [60]. Another mechanism attributing Kv11.1 its unique tropism for drugs is ’drug trapping‘, in which some drugs remain within the channel pore, prevented from moving out as a result of deactivation of the channel [43] before drug unbinding. This drug trapping mechanism could explain greater action potential prolongation and greater pro-arrhythmic risk to drug blockers with the same affinity and depends on the drug unbinding velocity relative to the deactivation of the channel.

However, direct Kv11.1 blockade has not been the only mechanism attributed to diLQTS. Drugs that block Kv11.1 can often block other cardiac ion channels that can either suppress or promote arrhythmogenic risk [43]. Drugs can have an unopposed Kv11.1 block (e.g., sotalol and dofetilide, which have high TdP risk) or can block Kv11.1 in addition to the blocking of inward currents (I_Ca-L_, I_NaL_), with a lower risk for TdP. Another suggested mechanism by which diLQTS can be induced is by a reduced hERG trafficking mechanism [44]. This mechanism causes a reduction in the number of Kv11.1 channels in the membrane as a result of defective trafficking of the channels from the endoplasmic reticulum. Drugs that exhibit this mechanism include fluoxetine, ketoconazole, arsenic trioxide among others [40,44].

#### 3.1.5. The Role of EADs in diLQTS

The real problem with QT interval prolongation is the increased risk for developing EADs. EADs are secondary depolarizations that occur during the plateau or repolarizing phase of the cardiac action potential as a result of prolonged repolarization, as is the case in the drug block of the Kv11.1 channel [61]. EADs are the key next step in the development of TdP after Kv11.1 blockade. They have been linked to the genesis of lethal arrhythmias, importantly TdP, but also ventricular tachycardia (VT) and ventricular fibrillation (VF), thus a lot of work has been done to elucidate the mechanisms by which EADs occur and, therefore, to find possible therapeutic targets for their prevention [62,63]. The development of EADs is not completely understood and much work is still missing in order to understand these rapid oscillatory membrane depolarizations. EADs are thought to be due to the reactivation of inward depolarizing currents (through L-type Ca^2+^ channels) during phase 2 or phase 3 before the membrane has fully repolarized [1]. However, EADs genesis does not always obey the intuitive notion that a net increase in the inward current is responsible (via increased inward current or decreased outward currents). Indeed, some experiments [62,63] have even shown contradictory results, in which ionic models that enhance inward currents reduce EADs occurrence. Consequently, EADs are commonly explained from a dynamical system point of view through the dual Hopf-Homoclinic bifurcation theory [62]. These bifurcation explanations are used in many nonlinear dynamical biological systems (e.g., cell cycle, circadian rhythms, glycolytic oscillations, among others) and they adequately explain the initiation and termination of EADs. The ionic determinants of the bifurcation hypothesis in cardiac repolarization have been investigated previously, and have shown that EADs are produced when the outward potassium current is delayed (as can happen in diLQT) during the Ca^2+^ channel window (where all L-type calcium channels are open, −10 mV to −45 mV) so that I_Ca-L_ reactivates and thus causes EADs [62]. There have been theories about how reducing the I_Ca-L_ window could prevent arrhythmogenesis in LQTS, and some cases have shown that shifting the steady-state inactivation curve of L-type Ca^2+^ channels could effectively suppress EAD [64,65]. Given the previously discussed cardiac heterogeneity, the myocytes with EADs serve as a depolarizing source to induce ventricular extrasystoles and thus premature ventricular contractions (PVCs). PVCs are a well-known ECG parameter preceding TdP (referred to as R-on-T) [66]. Therefore, the induction of EADs leads to the development of PVC and further increases the risk of developing TdP or other fatal arrhythmias. As discussed previously, drugs that can prolong the QT interval share a common mechanism by blocking the I_Kr_, rendering the ventricle susceptible to generate EADs and later TdP.

### 3.2. Drugs That Cause Acquired Long QT Syndrome

Due to the fatal outcomes, diLQTS can provoke, many lists have emerged trying to determine the relative risk of each drug to cause TdP or QT prolongation [20]. Perhaps the most up-to-date list appears on the CredibleMeds.org website, where the Arizona Center for Education and Research on Therapeutics (AZCERT) [24], based on an ongoing systematic analysis of available evidence, places drugs in different categories [67]. These categories are drugs with a known risk of TdP (drugs that prolong the QT interval and are associated with a known risk of TdP, even when taken as recommended), those with a possible risk of TdP (drugs that cause QT prolongation but lack evidence for a risk of TdP), those with a conditional risk of TdP (drugs associated with TdP but only under certain conditions of their use), and drugs to avoid in cLQTS (drugs that pose a high risk of TdP for patients with cLQTS) [67]. However, a ranked list of the drugs with a known risk of TdP cannot be made, because the risk depends on the drug use and the patient’s clinical characteristics. Drugs with a known risk for TdP are listed in Table 2, as well as their currently known mechanism to induce QT prolongation. As previously discussed, the common mechanism by which the majority of drugs produce QT prolongation and thus increased risk of TdP is through I_Kr_ blockade by Kv11.1 inhibition, as well as possible inhibition of Kv11.1 channel trafficking [40]. Nevertheless, apart from the pro-arrhythmic nature of these drugs, several risk factors need to be considered to adequately prevent aLQTS.

### 3.3. Risk Factors for Acquired Long QT Syndrome

TdP is a rare event usually occurring in patients with predisposing risk factors. The most known risk factors include female sex, electrolyte abnormalities, age > 65 years, structural heart disease, bradycardia, renal failure, concomitant use of two or more QT-prolonging drugs, genetic susceptibility, and diabetes, among others [34,87]. However, there is not a current risk factor score to categorize patients in high-, moderate-, or low- risk groups, although several studies have attempted to do so [87,88]. Table 3 elucidates known clinical risk factors for QT prolongation with high evidence according to CredibleMeds^®^ ongoing research [24].

#### 3.3.1. Drug Regimen and Interactions

Drug administration, dosage, and concomitant use of other drugs are important risk factors that can increase the risk of drug-induced arrhythmias in some patients. For most QT-prolonging drugs, arrhythmogenic risk increases as a function of plasma drug concentration, except for quinidine [10]. Therefore, any factor that increases the plasma drug concentration is a risk factor for aLQTS (e.g., advanced liver disease, overdose, renal dysfunction, rapid infusion by the intravenous route, or ingestion of specific CYP inhibitor drugs) [10]. Consequently, concomitant use of drugs that can prolong QT interval has been a problem, especially in the older population or in HIV patients who usually require multiple drug regimens. Table 4 puts into consideration strong CYP inhibitors (See Table S1 for a detailed list of these drugs) and correlates possible type X interactions with the respective drugs known to have QT prolongation and TdP risk. Type X interactions are those in which the administration of two medications interact with each other in a clinically significant manner. In these interactions, it is recommended to take action that may include aggressive monitoring, dosage changes, or choosing alternative agents [89]. Most of these interactions are due to strong inhibition of the drug’s corresponding CYP, increasing the plasma area under the curve (AUC) values, which as a result can dramatically increase the risk of QT prolongation and a fatal outcome. For instance, the interaction between amiodarone and voriconazole has shown to increase the mean QTc interval 49 ms from the baseline, since voriconazole increases amiodarone serum concentrations and amiodarone enhances the QT-prolonging effect of voriconazole [70]. Nevertheless, the exact risk for developing QT prolongation when using a specific combination of drugs is unknown. There have been some attempts to develop a clinical tool to determine the risk of QT prolongation when using two or more QTc-prolonging drugs with a known risk of TdP [90], but not without the need for further testing.

#### 3.3.2. Electrolyte Abnormalities

Hypokalemia is a strong independent risk factor for QT prolongation. The mechanism whereby hypokalemia causes QT prolongation does not obey the Nernst equation, where a decreased extracellular potassium concentration would cause a stronger gradient force, increasing potassium outflow and thus shortening the action potential duration and the QT interval [1]. Hypokalemia paradoxically causes QT prolongation because lower extracellular potassium concentration changes the rate at which Kv11.1 channels inactivate. There is a slowing of inactivation as a result of elevations in K+ concentrations and faster inactivation with lower K^+^ concentrations [1,64,92] Likewise, lowering extracellular potassium concentration increases the sensitivity of I_Kr_ to drug blocking [93].

Hypocalcemia has also been recognized as a risk factor for QT interval prolongation and TdP [94,95]. The calcium current is an important depolarizing current in the ventricular action potential. Its homeostasis determines the action potential duration, and—as previously discussed—it plays a role in the trigger of EADs. Low extracellular calcium decreases the influx of calcium to the cell, decreasing intracellular calcium, and indirectly decreasing the outward current via I_k(Ca)_ [96], prolonging phase 2 of the action potential. Some case reports [92] have pointed towards hypocalcemia-induced TdP, although this is rare.

On the other hand, magnesium is a cofactor for the Na^+^/K^+^ ATPase, facilitating the influx of potassium to the cells and thereby stabilizing the membrane potential [97]. Likewise, magnesium regulates a variety of potassium currents, such as I_Kr_ and I_to_, and also inhibits I_Ca-L_. However, hypomagnesemia is not a common risk factor for prolonged QT in some studies [87] as it is mostly arrhythmogenic in the presence of hypokalemia and bradycardia [96,98]. Despite the low frequency of hypomagnesemia-induced TdP, it is a relevant risk factor. In fact, AZCERT categorizes proton pump inhibitors (PPI) and many other drugs in the conditional risk of TdP category due to the hypomagnesemia- and hypokalemia-related risk of TdP [24]. Drugs that are associated with QT prolongation but only under certain settings (increased dose, comorbidities, electrolyte abnormalities) are presented by the AZCERT and can be seen in Table 5 along with the conditional risk factors that can result in TdP.

#### 3.3.3. Comorbidities

##### Heart Disease

In addition, stress cardiomyopathy with left ventricular apical involvement can also cause repolarization abnormalities that lead to VT/TdP development [99]. Even more worrisome are the arrhythmogenic properties that have been directly attributed to the COVID-19, with a pooled incidence of cardiac arrhythmia of 9.3% [100,101]. COVID-19 has been shown to cause cardiac injury reflected in elevated cardiac troponin I (cTnI) levels in approximately 14.7% of patients [100,101]. Myocardial damage might be a major factor involved in the enhanced arrhythmic risk in these patients [39], which added to the prolonging QT drugs used in COVID-19 treatment [35] (Table 6) could result in the development of a fatal arrhythmia. However, beyond the QT-prolonging drugs used in this disease, some cases [102] have emerged suggesting that COVID-19 could result in a further QT prolongation in patients with non-modifiable risk factors in the absence of high-risk drug therapy due to the exaggerated immune response to SARS CoV-2 [39]. 

Inflammation has been thought of as a novel QT-prolonging risk factor. Interleukin (IL-)6, tumor necrosis factor (TNF)-α, and IL-1 have been shown to prolong ventricular action potential by regulating ion channel expression in cardiomyocytes. IL-6 and TNFα in particular have been shown to decrease I_Kr_ current [103]. Accordingly, elevated TNF-α concentrations in patients with congestive heart failure are thought to contribute to QT prolongation and the increased risk for sudden cardiac death in these patients [104].

##### Impaired Renal Function

Chronic kidney disease (CKD) is another comorbidity commonly seen in hospitalized patients who develop diLQTS. The QT interval increases by 2.9 ms with each milligram increased in serum creatinine [64]. Approximately 56.9% of patients with CKD have a prolonged QT interval [105], and it is significantly prolonged with increasing CKD severity. The causes and mechanisms whereby patients with CKD have a prolonged QT include diabetes mellitus, which is responsible for 30–50% of all cases of CKD. Diabetic patients have a higher prevalence of aLQTS, and QT prolongation can even be used as a marker to detect the progression of albuminuria in patients with diabetic nephropathy [105]. Furthermore, diabetes may have a direct toxic effect in the Kv11.1 channels as the metabolic abnormalities it causes (hyperglycemia, hyperlipidemia) increases oxidative stress, with the creation of reactive oxygen species (ROS) which accumulate in the myocardium and cause direct channel dysfunction [64,106]. Likewise, people with CKD commonly have left ventricular function abnormalities, which—as previously discussed—is a risk factor for aLQTS. Moreover, urea, creatinine, parathyroid hormone, and homocysteine (all defined as ’uremic toxins‘) have all been associated with QT prolongation, due to increased ROS production, cardiac fibrosis, or myocardial hypertrophy [105]. Most importantly, electrolyte disorders and hemodialysis are a major contributor to the increased risk of QT prolongation in CKD patients, as hypokalemia and hypocalcemia are prevalent in this group of patients.

Although, as seen previously, there are drugs known to prolong the QT interval, the use of these drugs in the absence of other risk factors rarely causes a prolonged QT interval. In patients with no risk factors, the use of a QT-prolonging drug or a combination of these is acceptable in clinical practice [90]. Therefore, there is a need for a stratification scale to classify patients depending on their risk factors to develop diLQTS. Nonetheless, some algorithms have been developed. A study published in 2021 evaluating the performance of two previously developed algorithms in a substantial dataset found AUROC of 0.81 and 0.73 for the Bindraban and Berger algorithms respectively [90]. This study considered risk factors such as age, the use of loop diuretics, glomerular filtration rate, serum potassium, serum calcium, female gender, cardiac comorbidities, hypertension, diabetes mellitus and the use of QT-prolonging drugs. However, these tools need further improvement due to low discriminative ability.

## 4. Pharmacogenetic Determinants of QT Interval Prolongation

Pharmacogenetics is a discipline that studies the genetic basis of interindividual variability in drug response (i.e., effectiveness, adverse reactions, et cetera) concerning both pharmacokinetics and pharmacodynamics. Over the last few decades, there has been a growing interest in this field and its applications in personalized medicine. Many conditions and medications have been examined from the pharmacogenetics viewpoint and LQTS is not an exception to this. Recently, the attempted use of QT-prolonging drugs such as azithromycin and hydroxychloroquine for the treatment of COVID-19 highlighted the importance and risk-reducing potential of pharmacogenetics [107]. Moreover, the application of personalized medicine in LQTS is increasingly needed, primarily for prediction and prevention [20]. In this regard, a recent study weighed alleles of common genetic variants in multiple genes associated with baseline QT interval duration, based on GWAS data [108]. Such an approach works on the assumption that individual variants may have little influence on the phenotype of quantitative traits, whereas when occurring simultaneously in the same person, they have a greater effect (i.e., additive effect). Using this strategy, the authors demonstrated that this score, consisting of 61 single nucleotide polymorphisms (SNPs), was suitable for predicting the patient’s response to QT-prolonging drugs, as well as the risk of TdP [108].

The duration of the QT interval, as an electrocardiographic surrogate of ventricular repolarization, is a complex trait—i.e., one determined by genetic factors (e.g., common variants within ion-channel genes) and environmental factors, such as some drugs. Excluding patients with cLQTS, estimates of the heritability of this trait are around 35% [33]. Several genes associated with QT interval duration, including *NOS1AP* (encoding the nitric oxide synthase 1 adaptor protein) and some causative genes of cLQTS, have been uncovered, mainly by genome-wide association studies (GWAS) [109]. However, the genetic variants identified so far only explain a certain amount of the heritability, while a considerable proportion of it is considered as ‘missing heritability’ [110].

Genetic factors most certainly play a key role in each patient’s risk of developing diLQTS. The possible genetic influences on the susceptibility of this condition are various, for instance: common variants enhancing the QT-prolonging properties of some medications, rare variants within genes coding for ion channels, or variants affecting genes encoding proteins involved in drug kinetics (absorption, distribution, metabolism, and excretion) —also known as ADME genes [111,112]. As stated before, genes previously identified as the cause of cLQTS are associated with diLQTS as well. Furthermore, GWAS and whole-exome sequencing have been used in the search for genes implicated in the susceptibility for this entity, yielding several candidate genes as result—e.g., the solute carrier family members *SLC22A23* and *SLCO3A1*, the ceramide kinase-like gene (*CERKL*), the paladin, cytoskeletal-associated protein gene (*PALLD*), the bruno-like 4 gene (*BRUNOL4*), the neuregulin 3 gene (*NRG3*), the nucleotide-binding protein-like gene (*NUBPL*), the potassium voltage-gated channel subfamily E regulatory subunit 1 gene (*KCNE1*), and the succinate dehydrogenase complex assembly factor 3 (*SDHAF3*) [109,113,114]. A study evaluating the role of *NOS1AP* in calcium channel blocker-induced QT prolongation found that the rs10494366 GG genotype was associated with greater prolongation of the QTc interval in verapamil users, compared to the homozygous wild-type (TT) genotype [115]. Besides these findings, variants within genes encoding proteins involved in beta-adrenergic and sex hormones pathways may also be linked to a greater risk of diLQTS [116]. Given that research on this matter has largely focused on adults, it is not clear yet if diLQTS-related variants are the same in children and adolescents as in adult individuals; however, it is plausible to think that many of the polymorphisms identified in adults may also be relevant for the pediatric population [114].

ADME genes have been associated with the variability in the risk of QT prolongation. Phase I metabolism enzymes, namely the cytochrome P450 (CYP) family, play a central role in the metabolism of many drugs. In this case, CYP2D6 and CYP3A4/5 are responsible for the metabolism of many QT-prolonging drugs [114]. Therefore, patients with defective CYP2D6 and/or CYP3A4/5 (e.g., CYP2D6 ‘poor metabolizers’) might be at greater risk of QT prolongation as an adverse reaction to drugs metabolized by these enzymes. Some studies have linked *CYP2D6*, *CYP2C19*, *CYP2B6*, *CYP2C9*, and *CYP3A4/5* to diLQTS [109,114]. On the other hand, genes implicated in drug transport are also important in the relationship between some medications and a prolonged QT interval. Such is the case of the *ABCB1* gene, coding for the P-glycoprotein. Polymorphisms c.1236C > T, c.2677G > T, and c.3435C > T in this gene are the most studied ones in connection with the risk of diLQTS [109].

Some patients with cLQTS mutations may not show a significant QT interval prolongation without any triggering exposure (i.e., concealed cLQTS), but they are indeed at greater risk of diLQTS and drug-induced TdP [117]. As previously mentioned, *KCNH2*, whose mutations are responsible for LQT2, codes for the protein hERG. This protein is a subunit of a potassium voltage-gated channel that acts as a target of some drugs, including many QT-prolonging drugs. Several studies have identified an association between *KCNH2*, as well as other genes implicated in cLQTS (such as *KCNQ1* and *SCN5A*), and prolongation of the QT interval as a result of the intake of some medications [109,116]. Interestingly, diLQTS-related *KCNH2* mutations seem to be different from those described for cLQTS [109]. Regarding drug-induced TdP, up to 19% of these patients have mutations in the genes that account for cLQTS, possibly representing a form of incomplete penetrance of this syndrome that can go undetected until an environmental exposure (i.e., drug intake) [109,117]. Additionally, GWAS data has suggested that most of the risk of drug-induced TdP might not be explained by common variants [117].

It is widely known that ethnic origin influences drug response, which is largely due to different genetic backgrounds. Ethnicity is therefore a crucial factor to consider in the pharmacogenetic approach to diLQTS. However, existing literature on this matter is rather scarce. Little is known about how the QT interval behaves in normal conditions among individuals of different ethnicities, or how susceptibility to diLQTS varies between ethnic groups, and to what extent genes are responsible for these differences. The inclusion of some ethnic groups such as African Americans or Asians in thorough QT studies has not been as would be desired [109]. The duration of the QT interval at baseline seems to be similar among different ethnic origins, although there may be slight differences in some of them [118,119,120]. Conversely, multiple studies have found inter-ethnic differences in the distribution and allele frequencies of variants associated with the QT interval [109]. Furthermore, it has been shown that the risk of developing QT prolongation after acute drug (medication or illicit) overdose is greater among African American patients and lower in the Hispanic population [121]. Some clinical studies have also evidenced inter-ethnic differences concerning the QT prolongation risk to specific drugs (e.g., quinidine and some fluoroquinolones) [109,118,122,123].

Besides this, a sizable amount of evidence substantiates the existence of inter-ethnic differences in CYP genes, such as *CYP2D6*, as well as in genes such as *KCNH2* and other cardiac ion channel-encoding genes, thus supporting the role of ethnicity in diLQTS [118]. A recent study conducted in >3000 individuals from native and admixed Iberoamerican populations highlights the determining role of continental admixture on the frequencies of pharmaco-alleles of three CYP genes [124]. This work showed that the alleles CYP2D6*41 and CYP2C9*2 are positively associated with European ancestry, whereas CYP2D6*17 and CYP2D6*29 positively correlate with African ancestry [124]. All these four alleles reduce the activity of the corresponding CYP protein. It has been also observed that the variant rs4959235 in the aforementioned *SLC22A23* gene has a stronger effect on quetiapine-induced LQTS among Caucasian individuals than in African Americans, demonstrating that ethnic origin influences drug-gene interactions in diLQTS [125]. In addition to this, allele frequencies of SNPs associated with diLQTS/TdP (identified via GWAS) in the Latino/Admixed American population range from 2% (*PALLD* rs17054392) to 94% (*NRG3* rs4933824) (gnomAD v3.1 [126]), showing the important contribution of common variants in drug responses.

Continuing research in pharmacogenetics and pharmacogenomics concerning diLQTS will most certainly allow for a stratified, personalized approach in the treatment of many conditions for which QT-prolonging drugs are indicated, thus reducing the incidence of this adverse drug reaction. Such an approach must ponder both genetic and non-genetic variables in each patient. More studies are needed to better characterize the genetic substrate of interindividual variability in the risk of developing QT prolongation and TdP. In addition to this, advances in high-throughput sequencing techniques, allowing very short turnaround times and increasing reliability and affordability, may facilitate the incorporation of individualized genetic information into the clinical decision-making process when prescribing QT-prolonging drugs. Notwithstanding this, a careful cost-benefit analysis must be done before implementing these strategies in clinical practice. Likewise, thorough QT studies would also benefit from taking inter-ethnic differences and pharmacogenetic considerations into account during the process of developing and evaluating new drugs [116,118].

## 5. Conclusions

In conclusion, the molecular mechanisms by which drugs can prolong the QT interval are complex and common to many drugs from different classes. Kv11.1 is a widely studied potassium channel frequently implicated in this clinical phenomenon. However, a method that can effectively prevent a drug from causing QT interval prolongation, EADs, and—ultimately—TdP is lacking so far. Even though the risk factors for developing QT prolongation are well known, there is a need for a clinical scale or adequate stratification of patients at increased risk to develop diLQTS, including close monitoring of drug-drug interactions. Moreover, diLQTS is a complex entity, thus involving variation in many genes (i.e., polygenic). Although causative genes for cLQTS have also been associated with diLQTS, several other genetic loci have been reported as candidates for this disease. Both common and rare variants may contribute to the phenotype. Hence, increasing knowledge about the genetic basis of diLQTS should be translated into a personalized pharmacogenetic risk stratification approach when prescribing QT-prolonging drugs, considering genetic and non-genetic factors.

## Figures and Tables

**Table 1:** Summary of the molecular basis of congenital long QT syndromes and multiorgan syndromes including long QT syndromes.

Gene (Subtype) ^†^	Locus ^†^	Protein ^†^	Functional Effect ^†^	Mode of Inheritance ^†^	Frequency ^†^	Level of Evidence ^‡^
**Long QT syndrome (major)**						
*KCNQ1* (LQT1)	11p15.5	Kv7.1	Reduced I_Ks_	AD; AR	30–35%	Definitive
*KCNH2* (LQT2)	7q35-46	Kv11.1	Reduced I_Kr_	AD	25–30%	Definitive
*SCN5A* (LQT3)	3p21-p24	Nav1.5	Increased I_Na_	AD	5–10%	Definitive
**Long QT syndrome (minor)**						
*AKAP9* (AKAP9-LQTS)	7q21-q22	Yotiao	Reduced I_Ks_	AD	<1%	Disputed
*CACNA1C* (CACNA1C-LQTS)	12p13.3	Cav1.2	Increased I_Ca-L_	AD	<1%	Moderate
*CAV3* (CAV3-LQTS)	3p25	Caveolin 3	Increased I_Na_	AD	<1%	Limited
*KCNE1* (KCNE1-LQTS)	21q22.1	MinK	Reduced I_Ks_	AD	<1%	Limited
*KCNE2* (KCNE2-LQTS)	21q22.1	MiRP1	Reduced I_Kr_	AD	<1%	Disputed
*KCNJ5* (KCNJ5-LQTS)	11q24	Kir3.4	Reduced I_K,ACh_	AD	<1%	Disputed
*SCN4B* (SCN4B-LQTS)	11q23.3	Nav1.5 β4-subunit	Increased I_Na_	AD	<1%	Disputed
*SNTA1* (SNTA-LQTS)	20q11.2	Syntrophin-α1	Increased I_Na_	AD	<1%	Disputed
**Jervell and Lange-Nielsen syndrome**						
*KCNQ1* (JLNS1)	11p15.5	Kv7.1	Reduced I_Ks_	AR	Very rare	NA
*KCNE1* (JLNS2)	21q22.1	MinK	Reduced I_Ks_	AR	Very rare	NA
**Ankyrin-B syndrome**						
*ANKB* (ABS)	4q25-q27	Ankyrin B	Aberrant ion channel /transporter localization	AD	<1%	NA
**Andersen-Tawil syndrome**						
*KCNJ2* (ATS)	17q23	Kir2.1	Reduced I_K1_	AD	<1%	Definitive ^1^
**Timothy syndrome**						
*CACNA1C* (TS)	12p13.3	Cav1.2	Increased I_Ca-L_	Sporadic	Very rare	Definitive ^2^
**Recurrent infantile cardiac arrest syndrome**						
*CALM1*	14q24-q31	Calmodulin 1	Dysfunctional Ca^2+^ signaling	Sporadic	<1%	Definitive
*CALM2*	2p21	Calmodulin 2	Dysfunctional Ca^2+^ signaling	Sporadic	<1%	Definitive

Adapted from [31] (^‡^) and [33] (^†^). AD, autosomal dominant; AR, autosomal recessive; ABS, ankyrin-B syndrome; ATS, Andersen–Tawil syndrome; LQTS, long QT syndrome; TS, Timothy syndrome. ^‡^ Genetic evidence for cLQTS unless stated otherwise. ^1^ For Andersen–Tawil syndrome. Limited for cLQTS. ^2^ For Timothy syndrome. Moderate for cLQTS.

**Table 2:** Drugs with a known risk of TdP.

Drug Class (with Known Risk for TdP)	Drug	Molecular Mechanism for Prolonged QT Interval	Comments	Reference
Anticancer	Aclarubicin	-	Acute cardiotoxicity and heart failure coupled with QT prolontation in case reports and case series	[68]
	Arsenic trioxide	Inhibition of Kv11.1 trafficking	QT prolongation >500 ms (up to 40% of patients)	[35]
	Oxaliplatin	-	-	
	Vandetanib	I_Kr_ block	Incidence of QTc interval prolongation with 300 mg/day: 16.4% (all grades), 3.7% (high-grade)	[69]
Antiarrhythmic				
	Amiodarone	I_Kr_ block, binds to the closed state of the channel	Inactivation-removing mutations (S631A) reduce inhibitory effects on Kv11.1 channel	[70]
	Disopyramide	I_Kr_ block, I_Na_,	Dose-dependent prolongation	[71]
	Dofetilide	I_Kr_ block, I_Na-L_ augmentation	High risk of ventricular arrythmias, requires inpatient monitoring for approximately 3 days Adjust dose in CKD	[35]
	Dronedarone	I_Kr_ block	-	-
	Flecainide	I_Kr_ block Inhibition of CYP3A4	The risk of TdP correlates with the extent of prolongation of the QT interval.	[72]
	Hydroquinidine	I_Kr_ block	-	-
	Ibutilide	I_Kr_ block, I_Na-L_ augmentation		
	Nifekalant	-	-	-
	Procainamide	I_Kr_ block		
	Quinidine	I_Kr_ block, I_K1_, I_to_ inhibition		[71]
	Sotalol	I_Kr_ block, I_Na-L_ augmentation	-	-
Antihistamine				
	Astemizole	I_Kr_ block	High mortality related to concomitant administration with azole compounds	[73]
	Terfenadine	I_Kr_ block		[73]
Antibiotic				
	Azithromycin	I_Kr_ block, I_Na-L_ augmentation		
	Ciprofloxacin	I_Kr_ block		[73]
	Clarithromycin	I_Kr_ block Inhibition of CYP3A4	Greater risk of TdP at high doses and during IV administration	[73]
	Erythromycin	I_Kr_ block, I_Na-L_ augmentation Inhibition of CYP3A4	Greater risk of TdP at high doses and during IV administration	[73]
	Grepafloxacin			
	Gatifloxacin	I_K_ block, hERG block	Should be avoided in patients with risk factors of QT prolongation	[73]
	Levofloxacin	I_Kr_ block, hERG block		[74]
	Moxifloxacin	I_Kr_ block	Predictable 10–15 ms QTc prolongation	[1]
	Roxithromycin	I_Kr_ block		
	Sparfloxacin	Direct inhibition of hERG, I_Kr_ block	Potent compound blocking hERG and increases the instability of repolarization	[73]
Antianginal				
	Bepridil	-	-	-
Antimalarial				
	Chloroquine	I_Kr_ block, hERG block	Risk of death is proportional to drug exposure	[75]
	Halofantrine	I_Kr_ block		
	Hydroxychloroquine	I_Kr_ block, hERG block	Risk of TdP is dependent on dose	[76]
Antipsychotic				
	Chlorpromazine	I_Kr_ block, hERG block	Least potent blocking hERG K^+^ channels	[73]
	Chlorprothixene			
	Droperidol	I_Kr_ block	Leads to transient increases in QT and clinically significant QT prolongation in 1–9% of patients with other risk factors for QT prolongation	[77]
	Haloperidol	I_Kr_ block	Despite the relatively mild QTc prolongation associated with the oral and IM forms of haloperidol, this medication has clearly been linked to TdP	[77]
	Levomepromazine	-	-	-
	Levosulpiride	-	-	-
	Mesoridazine	hERG block	Prolongs the QT interval in a dose-dependent manner	[78]
	Pimozide	I_Kr_ block	Clear association with TdP	[77]
	Sertindole		The frequency of QTc intervals of 500 ms or longer is 3.1% to 7.8%	[72]
	Sulpiride	hERG block I_Kr_ block	Dose-dependent	[79]
	Sultopride			
	Thioridazine	I_Kr_ block, I_Na-L_ augmentation	Most associated with QTc prolongation among phenothiazines	[77]
Antidepressant				
	Citalopram	I_Kr_ block, and Inhibition of Kv11.1 trafficking	Prolongs the QT on the order of 10-20 ms	[77]
	Escitalopram	I_Kr_ block, and Inhibition of Kv11.1 trafficking	May carry some risk of mild QT prolongation, but not enough to be clinically significant	[77]
Anesthetic, general				
	Propofol	I_Kr_ block, Ito block	-	-
	Sevoflurane	I_Kr_ block, Ito block	Mean increase in maximal rate-corrected QT prolongation of 46 ms while patients are exposed to clinically relevant concentrations of the inhaled agent	[80]
Antiemetic				
	Domperidone	I_Kr_ block, hERG block	Dose-dependent risk factor of LQTS	[81]
	Ondansetron	I_Kr_ block	36 episodes of TdP were observed *p* < 0.02	[82]
Antifungal				
	Fluconazole	I_Kr_ block, and Inhibition of Kv11.1 trafficking	Implicated in three cases of QTc-interval prolongation when given alone or in combination with other drugs	[74]
	Pentamidine	Inhibition of Kv11.1 trafficking	Prolongs the cardiac action potential by blocking hERG trafficking and reduction of the number of functional hERG channels at the cell surface	[83]
Antiparasitic				
	Meglumine antimoniate	-	-	-
Antilipemic				
	Probucol	Inhibition of hERG trafficking		[73]
Cholinesterase inhibitors				
	Donepezil	I_Kr_ block, hERG block and trafficking inhibition	Long-term use of donepezil is associated with prolongation of the QT interval.	[84]
GI stimulant				
	Cisapride	hERG block	Risk of cisapride-related LQTS may be minimized by avoiding cofactors.	[73]
Local anesthetic				
	Cocaine	-	-	-
Muscle relaxant				
	Terodiline	-	-	-
Opioid agonist				
	Levomethadyl acetate		Higher potential to induce cardiac arrhythmias than does methadone in comparable dosages	[85]
Phosphodiesterase 3 inhibitor				
	Anagrelide	-	-	-
	Cilostazol	-	-	-
Psychedelic				
	Ibogaine	hERG block, calcium channel inhibition	Besides QT prolongation, a powerful psychoactive drug such as ibogaine may also lead to adverse cardiac effects related to its central nervous activity	[86]
Toxin				
	Cesium chloride	-	-	-
Vasodilator				
	Papaverine HCl	-	-	-

Adapted from: Known TdP risk drugs were taken from CredibleMeds^®^ (https://www.crediblemeds.org/, accessed on 1 February 2021) [24]. CKD, chronic kidney disease.

**Table 3:** Clinical factors associated with prolonged QTc.

System	Risk Factor
Autonomic nervous system	Pheochromocytoma Head-up-tilt Pure autonomic failure Sleep deprivation
Cardiovascular disease	Bradycardia Stress cardiomyopathy Stroke Aortic stenosis
Electrolyte disorders	Hypokalemia Hypomagnesemia Hypocalcemia Gitelman syndrome Blood transfusion
Endocrine disorders	Hypothyroidism Hyperparathyroidism
Environmental effects	Hypothermia Carbon monoxide Grapefruit juice Synthetic cannabinoids
General	Female sex, age > 65,
Inflammation/auto-immune	Rheumatoid arthritis Celiac disease Ankylosing spondylitis
Miscellaneous	Genotypic association Propionic academia Liquid protein diet Sickle cell disease

Table adapted from CredibleMeds^®^ list of clinical factors associated with prolonged QTc, only those with high quality evidence were taken into account and listed in the table [24].

**Table 4:** Known high TdP risk drugs and CYP strong inhibitors interactions.

CYP Metabolizer	Drug with a Known TdP Risk	Type X Interactions When Combined with (High Risk of QT Prolongation)
**CYP3A4**	Amiodarone	Voriconazol
		Indinavir
		Ritonavir
		Saquinavir
		Clarithromycin
	Disopyramide	Itraconazol
		Clarithromycin
		Ketoconazol (systemic)
		Saquinavir
		Telithromycin
	Dofetilide	Clarithromycin
		Itraconazole
		Ketoconazole
	Dronedarone	Clarithromycin
		Ceritinib
		Indinavir
		Itraconazole
		Nefazodone
		Ritonavir
		Saquinavir
		Telithromycin
		Voriconazole
	Ibutilide	Clarithromycin
	Quinidine	Clarithromycin
		Itraconazol
		Ketoconazol
		Ritonavir
		Saquinavir
		Voriconazole
	Azithromycin	Certinib
		Clarithromycin
		Saquinavir
		Voriconazole
	Clarithromycin	Saquinavir
	Erythromycin	Saquinavir
	Cloroquine	Ceritinib
		Clarithromycin
		Saquinavir
		Voriconazole
	Chlorpromazine	Certinib Saquinavir
		Voriconazol
		Clarithromycin
	Haloperidol	Saquinavir
	Levomepromazine	Ritonavir Saquinavir
	Escitalopram	-
	Ondansetron	-
	Cisapride	Ceritinib
		Clarithromycin
		Indinavir
		Itraconazole
		Ketoconazole
		Nefazodone
**2D6**	Flecainide	
	Ibutilide	Quinidine
	Procainamide	Quinidine
	Chloroquine	-
	Hydroxychloroquine	-
	Chlorpromazine	-
	Haloperidol	-
**2C19**	Citalopram	-
	Escitalopram	-
	Chlorpromazine	-

The drugs metabolized by a given CYP were crossed with the drugs known inhibit the respective CYP (See Supplementary Table S1) taken from [91]. Known TdP risk drugs were taken from CredibleMeds^®^ list of known risk of TdP drugs [24]. Drug interactions were analyzed for the potential drug interactions using Lexi-Interact™ Online available on the website www.uptodate.com (accessed on 10 June 2021) [89].

**Table 5:** CredibleMeds^®^ list of drugs with a conditional risk for TdP and risk factors.

Drug	Increased Risk in the Setting of					Use Causes Hypokalemia and/or Hypomagnesemia	Reduces Elimination of a QT/TdP Drug	Use in cLQTS
	Bradycardia	Hypokalemia/ Hypomagnesemia	Excesive Dose	Use with Concomitant QT/TdP Drug	Impaired Drug Elimination			
Abiraterone						X		
Amantadine	X	X	X	X				
Amisulpride		X	X	X				
Amitriptyline	X	X	X	X	X			
Amphotericin B				X		X		
Amsacrine						X		
Atazanavir				X				
Bendroflumethiazide				X		X		
Chloral hydrate			X					
Cimetidine	X	X		X			X	
Clomipramine		X	X	X				
Diphenhydramine			X					
Doxepin		X	X	X	X			
Eperisone			X					
Esomeprazole				X		X		
Famotidine		X	X	X	X			
Fluoxetine	X	X	X	X	X			
Fluvoxamine		X	X	X			X	
Furosemide				X		X		
Galantamine	X	X	X	X				
Garenoxacin		X		X				
Hydrochlorothiazide						X		
Hydroxyzine	X	X	X	X				
Indapamide		X		X				
Itraconazole				X			X	
Ivabradine	X	X		X	X			
Ketoconazole	X			X			X	
Lansoprazol				X		X		
Loperamide			X		X			
Metoclopramide	X	X		X				X
Metolazone		X	X	X		X		
Metronidazole		X		X				
Nelfinavir	X	X		X				
Olanzapine		X		X				
Omeprazole				X		X		
Pantoprazole				X		X		
Paroxetine							X	
Piperacilin/tazobactam	X	X		X		X		
Posaconazole	X	X	X	X			X	
Propafenone	X		X	X				
Quetiapine	X	X	X	X	X			
Quinine sulfate		X	X	X				
Ranolazine	X	X		X	X			
Risperidone	X	X	X	X				
Sertraline	X	X		X			X	
Solifenacin	X	X		X				
Telaprevir				X			X	
Torsemide				X		X		
Trazodone	X	X	X	X				
Voriconazole		X		X			X	
Ziprasidone	X	X	X	X				

Adapted from: CredibleMeds^®^ list of drugs with a conditional risk for TdP [24].

**Table 6:** QT prolonging drugs used in COVID-19 infection.

Antibiotics	Cloroquine/Hydroxycloroquine Macrolides Quinolones
Antiviral agents	Lopinavir/Ritonavir Favipiravir Tocilizumab Fingolimod
Anesthetics	Propofol
Antiemetics	Domperidone
Antiarrythmics	Class IA Class III

## Supplementary Materials

The following are available online at https://www.mdpi.com/article/10.3390/ijms22158090/s1.
