# Supplementary material for: Genetic and Molecular Aspects of Drug-Induced QT Interval Prolongation
Source: Int J Mol Sci. 2021 Jul 28;22(15):8090. doi: 10.3390/ijms22158090 (PMC8347245; doi:10.3390/ijms22158090)
Supplement: Supplementary file 1 [file ijms-22-08090-s001.zip › ijms-1213498-supplementary.pdf]

# Supplementary Material

**Table S1**. Strong CYP inhibitors

| **CYP Metabolizer** | **Strong CYP Inhibitors** |
|---|---|
| 3A4 | Clarithromycin<br>Indinavir<br>Itraconazol<br>Ketoconazol<br>Nefazodone<br>Nelfinavir<br>Ritonavir<br>Saquinavir<br>Ceritinib<br>Telithromycin<br>Voriconazole |
| 2D6 | Bupropion<br>Cinacalcet<br>Fluoxetine<br>Paroxetine<br>Quinidine |
| 2C19 | Fluvoxamine<br>Ticlopidine |
| 1A2 | Ciprofloxacin<br>Fluvoxamine |
